# Supplementary material for: The morphospace of the brain-cognition organisation
Source: Nat Commun. 2024 Sep 30;15:8452. doi: 10.1038/s41467-024-52186-9 (PMC11443123; doi:10.1038/s41467-024-52186-9)
Supplement: Supplementary file 1 — Supplementary Information [file 41467_2024_52186_MOESM1_ESM.pdf]

SUPPLEMENTARY MATERIAL FOR

The morphospace of the brain-cognition organisation

Valentina Pacella[abc]*, Victor Nozais[bc], Lia Talozzi[dbc], Majd Abdallah[bcef], Demian Wassermann[ef], Stephanie J Forkel[cghi], Michel Thiebaut de Schotten[bc]*

[a] Scuola Universitaria Superiore IUSS, Pavia, 27100, Italy.

[b] Groupe d'Imagerie Neurofonctionnelle, Institut des Maladies Neurodégénératives-UMR 5293, CNRS, CEA, University of Bordeaux, Bordeaux, 33076, France.

[c] Brain Connectivity and Behaviour Laboratory, Sorbonne Universities, Paris, 75006, France.

[d] Department of Neurology and Neurological Sciences, Stanford University School of Medicine, Stanford, California, 94305, USA

[e] MIND team, Inria Saclay Île-de-France, Université Paris-Saclay, Inria, 1 Rue Honoré d'Estienne d'Orves, Palaiseau, 91120, Ile-de-France, France.

[f] Neurospin, CEA, Gif-sur-Yvette, 91191, Ile-de-France, France.

[g] Donders Centre for Cognition, Radboud University, Thomas van Aquinostraat 4, 6525 GD Nijmegen, the Netherlands

[h] Centre for Neuroimaging Sciences, Department of Neuroimaging, Institute of Psychiatry, Psychology and Neuroscience, King's College London, London, SE5 8AF, United Kingdom.

[i] Departments of Neurosurgery, Technical University of Munich School of Medicine, Munich, 81675, Germany.

* Corresponding authors: valentina.pacella.90@gmail.com, michel.thiebaut@gmail.com

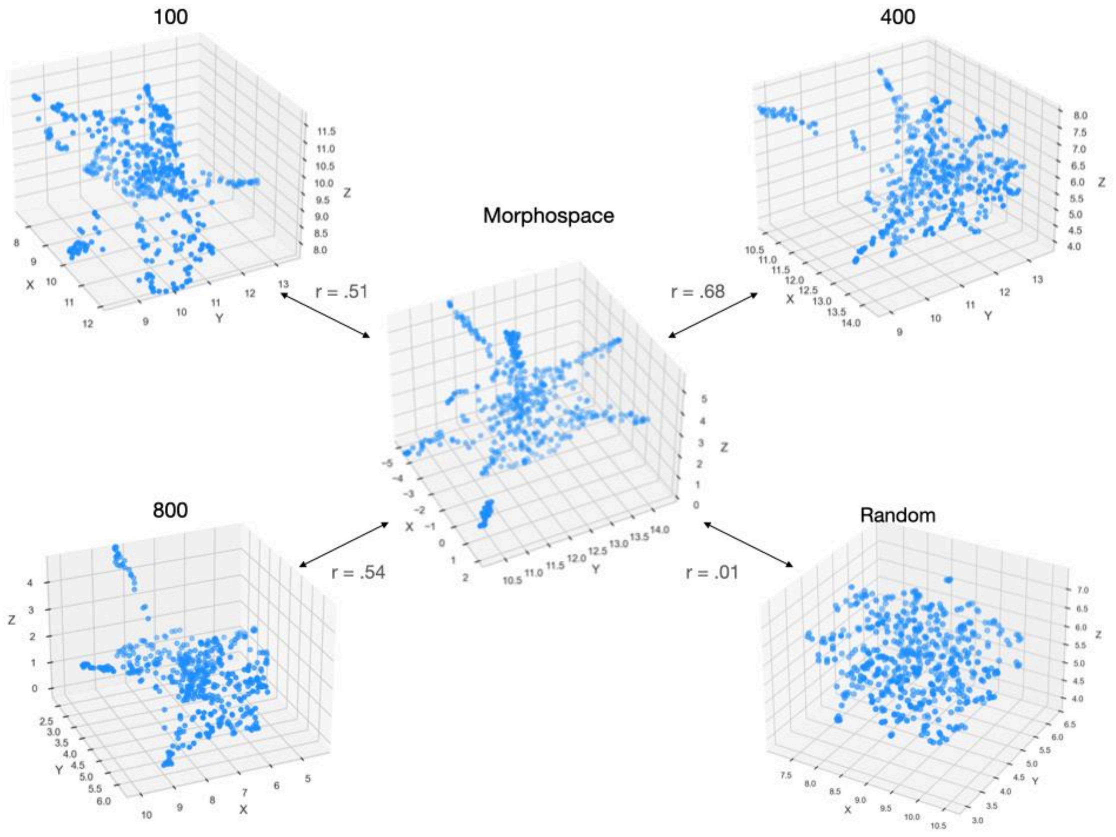

Supplementary Figure 1. Replication of the morphospace in four additional parcellation approaches. Pearson's r of the comparison between each additional parcellation and the morphospace is indicated for the 100 (top left), 400 (top right), 800 (bottom left) and a control random parcellation (i.e. null model, bottom right).

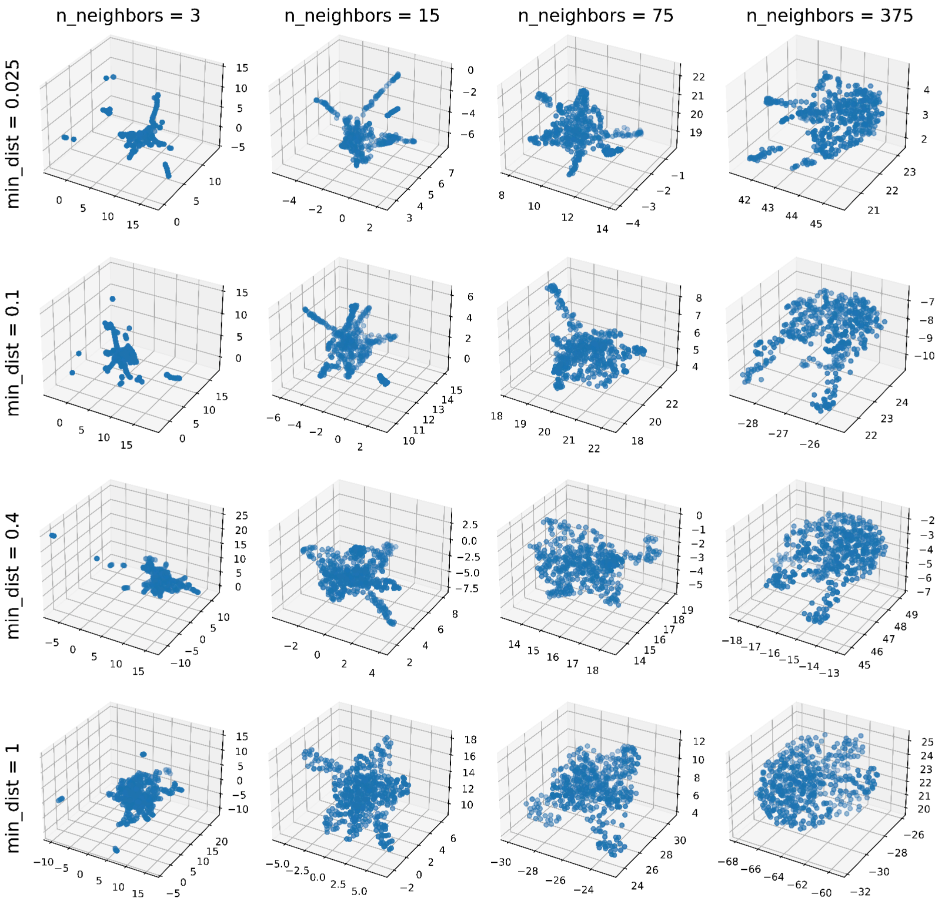

Supplementary Figure 2. Representation of the morphospace embedding with different n neighbours and minimum distance values. Higher values average the manifold largely across the data and vastly distribute the data in the low embedding. Extremely low values result in spurious clusters of connected neighbours. Given that the neuron-shaped architecture is stable across low to medium values and that there is no goal standard in the finer manifold/global structure trade-off, we opted to rely on the low default metrics that reveal the data manifolds maintaining the global structure without spurious neighbouring connections. Points represent the 506 meta-analytic maps.

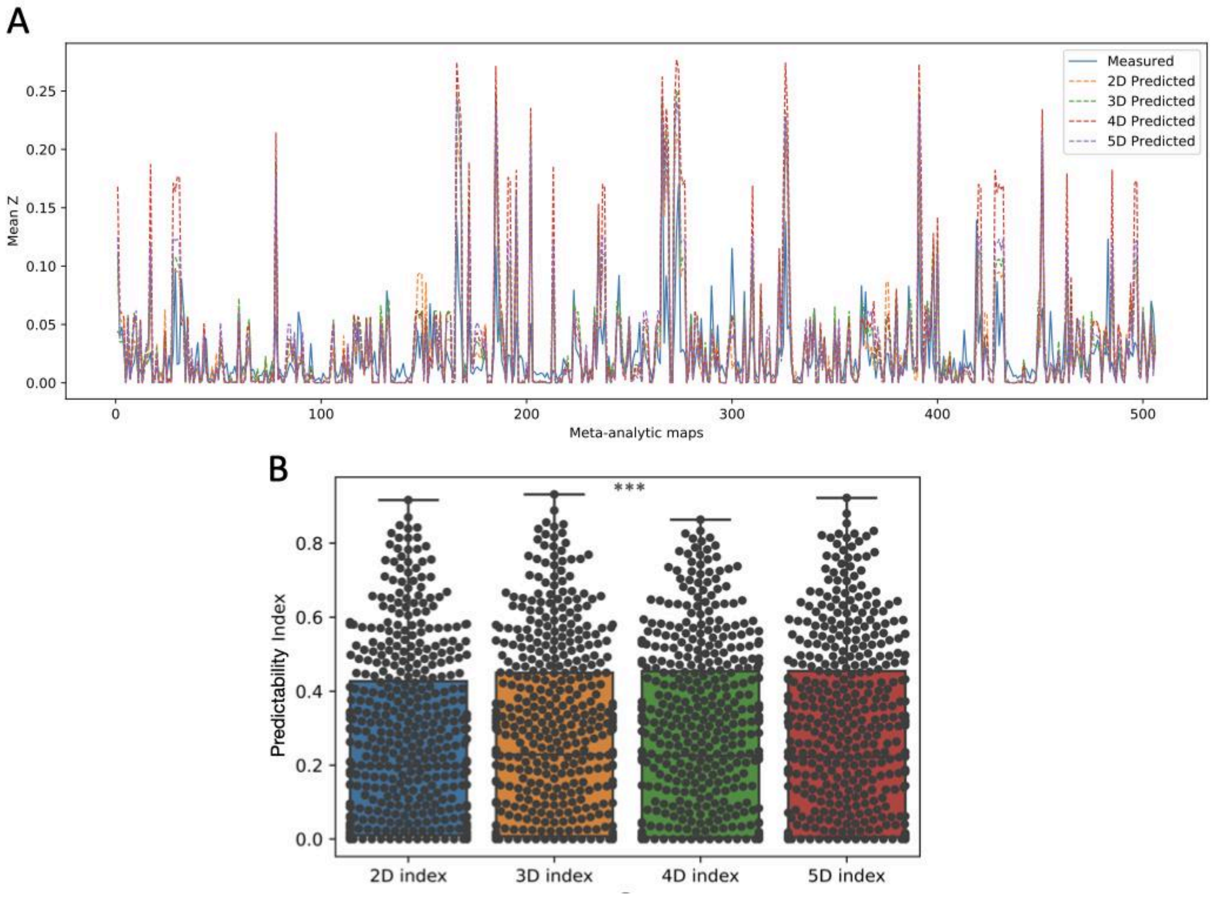

Supplementary Figure S3. Comparison between versions of the morphospace built in 2, 3, 4, and 5 dimensions. (a) The distribution of the MAE between measured and predicted maps indicates that the 3D predictions are more precise than the 2D ($z = 4.37$; $p<.001$) and 5D ($z = 5.18$; $p<.001$). (b) The difference in predictability index between the different dimensions indicates that the 3D predictability index is higher than the 4D ($z = 3.29$; $p<.001$), and the 2D ($z = 9.63$; $p<.001$). ***: $p<.001$. Lines, boxes, whiskers and dots represent the median, quartiles, distribution, and observations (506 meta-analytc maps).

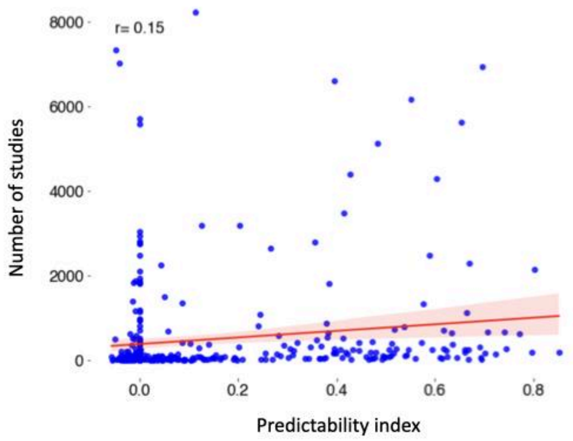

Supplementary Figure 4. The association of the predictability index the number of terms aggregated in each out-of-sample meta-analytic map. The low Pearson's correlation (r = 0.15) between the predictability index (x-axis) and the number of terms aggregated in each map (y-axis) reveals that the index is not associated with the magnitude of data used for each meta-analysis. Datapoints representing the 888 meta-analytic maps are represented in blue, and the regression line is indicated in red. r: Pearson's regression coefficient.

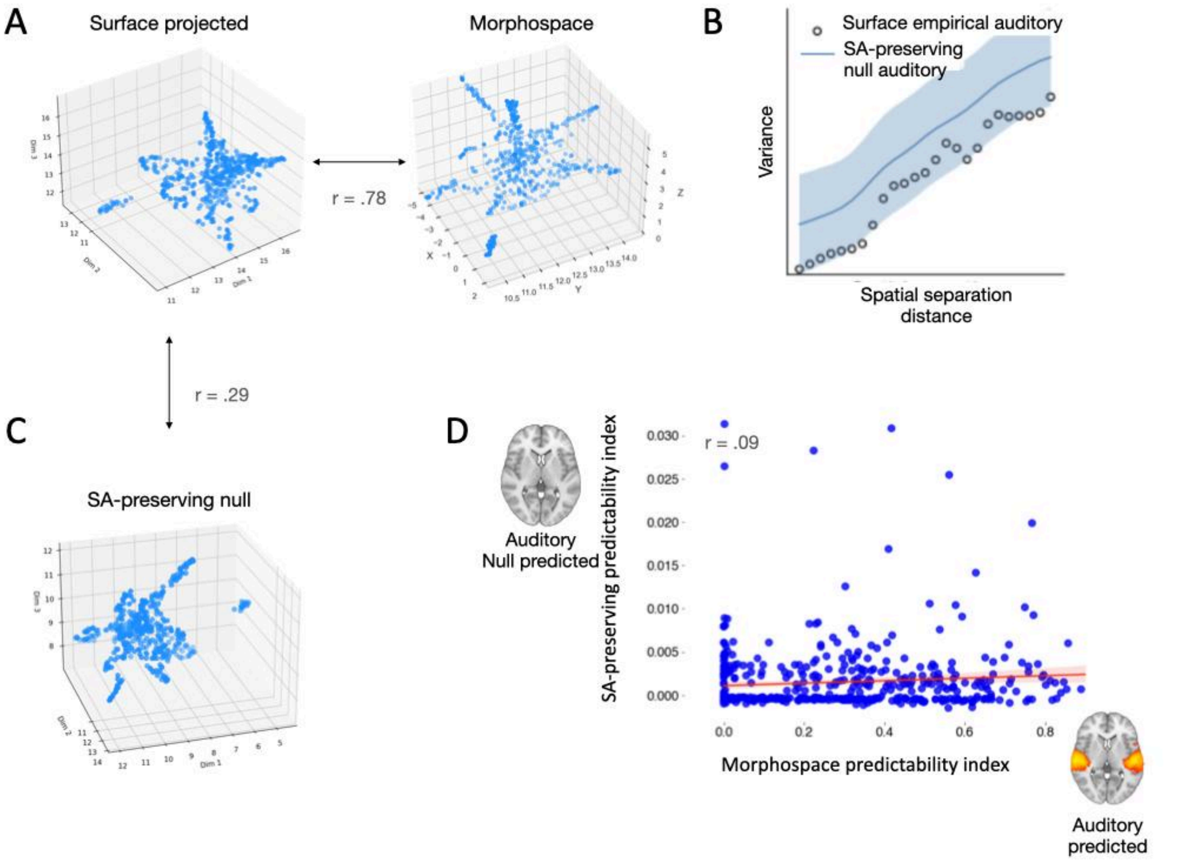

Supplementary Figure S5. Robustness of results against a spatial autocorrelation-preserving null model. a) Pearson's correlation of the comparison between the 400 parcels resolution of the Schaefer and colleagues atlas (2018) applied to the maps' surface projection and the original morphospace. b)The plot shows the distribution similarity between the surface-projected empirical and SA-preserving null versions an example meta-analytic map (auditory). c) Pearson's r of the comparison of the Euclidean distances between the surface-projected empirical and SA-preserving null maps, both parcellated via Schaefer and colleagues' atlas (2018). d) Pearson's r of the comparison between predictability indices obtained from the predicted 506 meta-analytic maps and the predicted SA-preserving null maps. Datapoints representing the 506 meta-analytic maps are represented in blue, and the regression line is indicated in red. The axial slices next to the x and y axes represent the example version of the auditory map predicted from the original morphospace and the SA-preserving null space, respectively. Dim: dimension. SA: spatial autocorrelation.

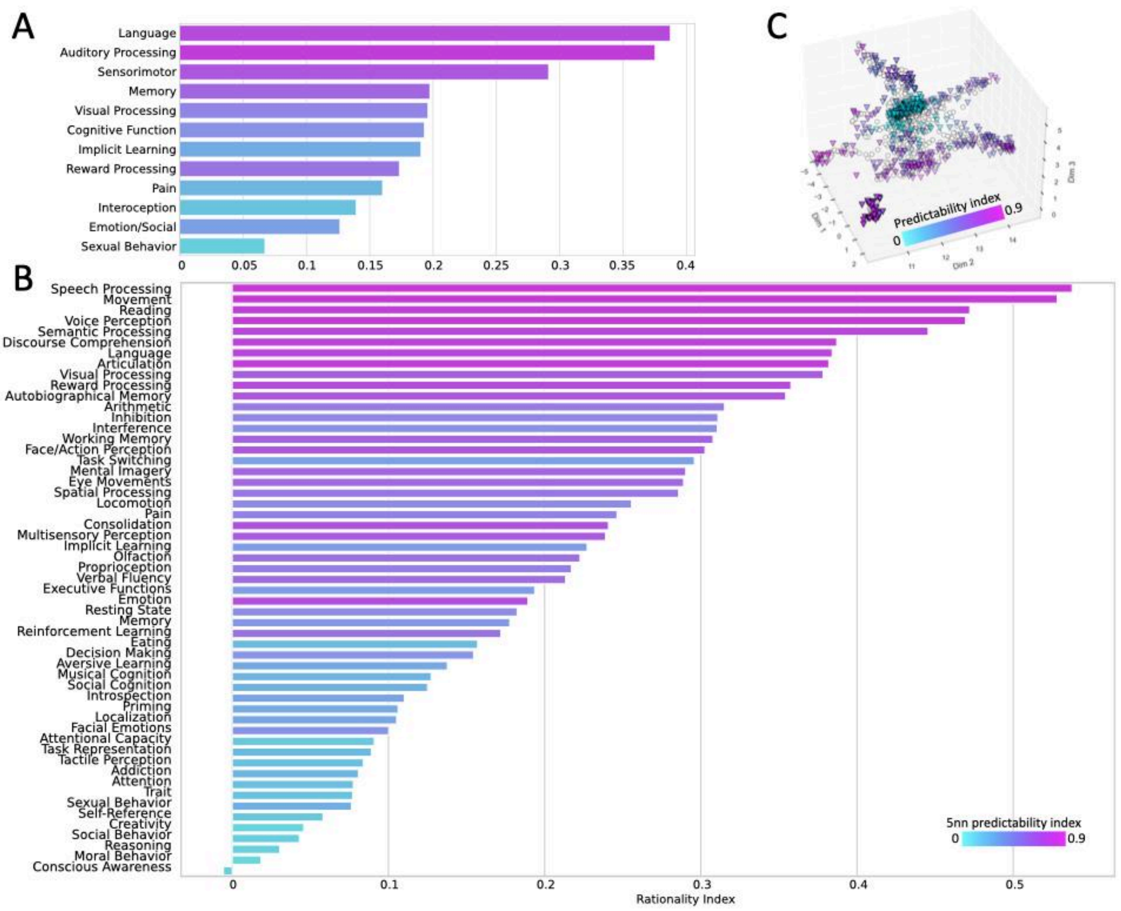

Supplementary Figure S6. predictability index of 888 Neuroquery meta-analytic maps. Topic modelling allowed for (a) 12 broader and (b) 55 finer clusters summary of the predictability index of (c) the meta-analytic maps colocalised onto the morphospace. Bar colours indicate the predictability index of their five nearest neighbours (5nn) in the morphospace. Triangles indicate each new map's coordinate in the morphospace, while transparent circles indicate the morphospace meta-analytic maps' location.

Table S1 Features of the new task-related activation maps.

Map	Type of map	Reference	Task	Contrast	Results
Abstract Words	T-map	Pauligk et al., 2019[1]	A delayed lexical decision task required indicating if a visually presented stimulus was a word or a pseudoword by left and right button presses.	Abstract VS. concrete words	Inferior frontal, superior and middle temporal cortices
Emotional Words	T-map	Pauligk et al., 2019[1]	Same as above.	Emotional VS. neutral words	Superior and medial frontal, cingulate cortices, middle temporal gyrus and amygdala, precuneus
Concrete Words	T-map	Pauligk et al., 2019[1]	Same as above	Concrete VS. abstract words	Superior and middle frontal gyri, medial temporal and calcarine cortices
Acute Fear	T-map	Hudson et al., 2020[2]	Participants watched feature-length horror movies.	Joint analysis of jump-scare events	Cingulate, Temporal, Insular cortices, Amygdala and Thalamus
Congruent Movement	T-map	Limanowski and Friston, 2020[3]	Participants control a virtual hand and are asked to match the movement with their own or the virtual hands.	Congruent VS. incongruent movement	Superior, middle temporal and postcentral gyri, somatosensory cortex

Visuo-propri oception	T-ma p	Limanowski and Friston, 2020[3]	Same as above	Visuo-propri oception of Congruent VS. Incongruent movement	Bilateral temporal and left secondary somatosensory cortices
Danger Expectance	T-ma p	Suarez-Jimen ez et al., 2018[4]	Activity differences during stationary periods of the threat learning task after picking flowers predicting either danger or safety.	Danger VS. safe	caudate, dACC, insula and midbrain
Friends Ownership	T-ma p	Lockwood et al., 2018[5]	Associative learning task foster learning about fractal images that belonged to participants, their best friend, or a stranger. Ownership associative strength (OAS) between picture and label at the time of the picture (the strength of ownership) and the size of the ownership prediction error (OPE) at the time of the outcome.	Friends_OAS	Ventromedial prefrontal and cingulate cortices, middle temporal gyrus and medial temporal cortex.
Friends Prediction	T-ma p	Lockwood et al., 2018[5]	Same as above.	Friends_OPE	Caudate, putamen, globus pallidum, left inferior temporal gyrus.

Ingroup Prediction Errors	T-map	Zhou et al., 2021[6]	Participants expected to receive painful shocks but were saved from pain by different ingroup or outgroup members in 75% of all trials. Initial ingroup bias in impression ratings was significantly reduced over the course of learning (prediction errors).	Ingroup vs. outgroup prediction errors	Inferior parietal lobule and anterior insula.
Latent Group	T-map	Lau et al., 2020[7]	Participants are asked to report their position on a political issue. They then learned the positions of three other hypothetical participants (A, B and C) on the same issue (trial-by-trial dyadic similarity learning). After repeating this procedure for eight different issues, the volunteers had to decide whether they would align with A or with B on a 'mystery' political issue (latent structure learning is influenced by C views).	Latent structure learning	Right Anterior Insula and Inferior Frontal Gyrus
Social Dyadic Similarity	T-map	Lau et al., 2020[7]	Same as above.	Trial-by-trial dyadic similarity index	Pregenual Anterior Cingulate
Learning through verification	T-map	Berens et al., 2018[8]	Participants have to associate unfamiliar objects with obscure pseudowords. Learning through verification model predicts that the representations rapidly change from being	Whole-brain searchlight representational similarity analysis on learning through	Left hippocampus

			equally similar to all others before they have been learnt to being dissimilar after learning.	verification model	
Memory Integration	T-map	van Kesteren et al., 2020[9]	Participants learn combination of pseudoword and scene (AB association) and object (AC association) so that B and C were linked via A in a congruent (known) or incongruent (unknown) manner.	AB encoding	Middle and inferior temporal gyri and cuneus
Congruency	T-map	van Kesteren et al., 2020[9]	Same as above	Correct associations	Medial prefrontal cortex, hippocampal and parietal cortices
Speech	T-map	Steiner et al., 2021[10]	Participants discriminate nonverbal (non-speech) and speech-based voice and non-voice (natural, artificial) sounds	Speech VS. non-voice	Left anterior, middle and posterior superior temporal gyrus, posterior superior temporal sulcus
Voice	T-map	Steiner et al., 2021[10]	Same as above	Voice VS. non-voice	Left middle and posterior superior temporal and right middle superior temporal sulcus
Touch	T-map	Suvilehto et al., 2021[11]	Touch is delivered by confederates on the upper thigh of the participants	Touch stimulation VS. Baseline	Insular, primary and secondary somatosensory cortex

Relationship between cognitive domains branches

The neuron-shaped architecture of the morphospace clusters cognitive domains within each branch, with the position of each branch reflecting the relationship between the domains. For instance, the close position of the vision and attention branches with regard to others reflects the anatomical overlap between activations related to vision paradigms, from simple stimuli observation to eye-tracking paradigms, and with attentional networks[12]. Vision and action activations are also closely located in the morphospace, and their interaction is known to manifest as embodiment mechanisms (e.g. rubber hand illusion[13]). Motor cognition and somatosensory mechanisms are jointly recruited during a movement to ensure online control and the successful outcome of the performance[14]. Further, the clusterisation of the domains within the morphospace shows that the emotion and somatosensory domains are adjacents, reflecting bodily signal generation and processing of emotional responses[15-17]. Emotions and somatic responses guide decision-making[18] as confirmed by the proximity of Emotion and Decision-making within the morphospace. The joint contribution of learning and memory allows humans to orient in social experiences[19], thus their close clusterisation in the space. Contextualisation of memories occurs by assigning meaning and words to encoded items[20], and language has been ascribed as part of the working memory as the phonological loop component[21]. Accordingly, memory, language, and working memory have interrelated aspects and follow one another in the morphospace. The auditory cognition clusters far away from the other domains. The striking difference in the anatomical pattern of auditory-modality fMRI task opens further queries on the possible influence of stimuli modality in activation studies.

Brain structures of the predictability *map*

The cerebral regions associated with high predictability indices exhibit a strong correlation with the gradients that explain overall brain activity in particular areas of auditory and motor processing[22]. The superior temporal cortex has been shown to contribute to auditory cognition[23] and processing of the object's spatial features[24], while medial temporal cortices such as the rhinal cortex, hippocampus, and amygdala play a role in memory[25-28] and stimuli representation (e.g. objects, faces, and scenes)[29]. The premotor cortex contributes to action planning[30] and speech[31], while the FEF and PEF are involved in visual target detection[32,33]. An extensive range of functions for the implementation of voluntary action, such as timing, sensory predictions, sequence implementation, and inhibition of concurrent movements, involve the SMA and pre-SMA areas[34-36]. Finally, the involvement of Broca's area as a hub of the language network in the brain has been extensively confirmed by the literature since its first description by Broca in 1861[31,37,38]. Prediction, learning and reward mechanisms emerge from the activity of subcortical structures such as the basal ganglia and its connections[39,40] as well as the medial temporal lobe structures[41].

Replication of the morphospace *space architecture in 2021 dataset*

In order to assess the morphospace reliability in terms of clustering cognitive domains and their spatial positioning, the Euclidean distances between the morphospace maps were compared to the distances in a three-dimensional space built using the updated 2021 version of the 2017 meta-analytic maps. A total of 506 maps were obtained from the Neurosynth repository and matched with the 2017 dataset terms. No thresholding was applied to the 2021 meta-analytic maps as the newer version of Neurosynth automatically corrects for multiple comparisons by applying a threshold of $z \geq 3.4$. The maps underwent parcellation using the Glasser and colleagues[42] and AAL3[43,44] atlases delineated by our group.

The Uniform Manifold Approximation and Projection (UMAP[45]) algorithm was applied to reduce the dimensionality of the parcelled 2021 meta-analytic dataset in a three-dimensional space, and UMAP default parameter values were used. Specifically, the algorithm used the information of 15 local neighbours to learn the manifold structure of the data points; 0.1 minimum distance was allowed by the algorithm to pack the data; the Euclidean metric was used for the data embedding.

Using Python (https://github.com/vale-pak/BCS.git), the Euclidean distances between the 2021 three-dimensional space maps were computed and subsequently compared with those of the 2017 morphospace meta-analytic maps. Pearson's correlations revealed a positive correlation ($r = 0.53$), affirming that the cognitive domain clusterisation and positioning observed in 2017 can be reliably replicated in later versions of the dataset.

Supplementary References

1. Pauligk, S., Kotz, S. A. & Kanske, P. Differential Impact of Emotion on Semantic Processing of Abstract and Concrete Words: ERP and fMRI Evidence. *Sci. Reports* **9**, 1–13 (2019).
2. Hudson, M. *et al.* Dissociable neural systems for unconditioned acute and sustained fear. *Neuroimage* **216**, (2020).
3. Limanowski, J. & Friston, K. Attentional Modulation of Vision Versus Proprioception during Action. *Cereb. Cortex* **30**, (2020).
4. Suarez-Jimenez, B. *et al.* Linked networks for learning and expressing location-specific threat. *Proc. Natl. Acad. Sci. U. S. A.* **115**, 1032–1040 (2018).
5. Lockwood, P. L. *et al.* Neural mechanisms for learning self and other ownership. *Nat. Commun.* **9**, 1–11 (2018).
6. Zhou, Y. *et al.* Learning from ingroup experiences changes intergroup impressions. Preprint at https://www.biorxiv.org/content/10.1101/2021.11.02.466926v1 (2021).
7. Lau, T., Gershman, S. J. & Cikara, M. Social structure learning in human anterior insula. *Elife* **9**, (2020).
8. Berens, S. C., Horst, J. S. & Bird, C. M. Cross-Situational Learning Is Supported by Propose-but-Verify Hypothesis Testing. *Curr. Biol.* **28**, (2018).
9. van Kesteren, M. T. R., Rignanese, P., Gianferrara, P. G., Krabbendam, L. & Meeter, M. Congruency and reactivation aid memory integration through reinstatement of prior knowledge. *Sci. Reports* **10**, 1–13 (2020).
10. Steiner, F., Bobin, M. & Frühholz, S. Auditory cortical micro-networks show differential connectivity during voice and speech processing in humans. *Commun. Biol.* **4**, 801 (2021).
11. Suvilehto, J. T., Renvall, V. & Nummenmaa, L. Relationship-specific Encoding of Social Touch in Somatosensory and Insular Cortices. *Neuroscience* **464**, (2021).
12. Corbetta, M. *et al.* A Common Network of Functional Areas for Attention and Eye Movements. *Neuron* **21**, 761–773 (1998).
13. Ehrsson, H. H., Spence, C. & Passingham, R. E. That's my hand! Activity in premotor cortex reflects feeling of ownership of a limb. *Science* **305**, 875-877 (2004).
14. Friston, K. The free-energy principle: a unified brain theory? *Nat. Rev. Neurosci.* **11**, 127–138 (2010).
15. Damasio, A. R. The somatic marker hypothesis and the possible functions of the prefrontal cortex. *Philos. Trans. R. Soc. London. Ser. B Biol. Sci.* **351**, 1413–1420 (1996).
16. Bufalari, I., Aprile, T., Avenanti, A., Di Russo, F. & Aglioti, S. M. Empathy for pain and touch in the human somatosensory cortex. *Cereb. Cortex* **17**, 2553–2561 (2007).
17. McGlone, F., Wessberg, J. & Olausson, H. Discriminative and affective touch: sensing and

feeling. *Neuron* **82**, 737–755 (2014).
18. Bechara, A., Damasio, H. & Damasio, A. R. Emotion, Decision Making and the Orbitofrontal Cortex. *Cereb. Cortex* **10**, 295–307 (2000).
19. Leblanc, H. & Ramirez, S. Linking Social Cognition to Learning and Memory. *J. Neurosci.* **40**, 8782 (2020).
20. Tulving, E., Kapur, S., Craik, F. I., Moscovitch, M. & Houle, S. Hemispheric encoding/retrieval asymmetry in episodic memory: positron emission tomography findings. *Proc. Natl. Acad. Sci. U. S. A.* **91**, 2016–20 (1994).
21. Baddeley, A. D. & Hitch, G. Working Memory. *Psychol. Learn. Motiv. - Adv. Res. Theory* **8**, 47–89 (1974).
22. Margulies, D. S. *et al.* Situating the default-mode network along a principal gradient of macroscale cortical organization. *Proc. Natl. Acad. Sci. U. S. A.* **113**, 12574–12579 (2016).
23. Jackson, R. L., Bajada, C. J., Rice, G. E., Cloutman, L. L. & Lambon Ralph, M. A. An emergent functional parcellation of the temporal cortex. *Neuroimage* **170**, 385–399 (2018).
24. Karnath, H. O. New insights into the functions of the superior temporal cortex. *Nat. Rev. Neurosci.* **2**, 568–576 (2001).
25. McGaugh, J. L. Memory--a Century of Consolidation. *Science* . **287**, 248–251 (2000).
26. Dudai, Y., Karni, A. & Born, J. The Consolidation and Transformation of Memory. *Neuron* **88**, 20–32 (2015).
27. Binder, J. R. & Desai, R. H. The neurobiology of semantic memory. *Trends Cogn. Sci.* **15**, 527–536 (2011).
28. Squire, L. R., Stark, C. E. L. & Clark, R. E. The medial temporal lobe. *Annu. Rev. Neurosci.* **27**, 279–306 (2004).
29. Robin, J., Rai, Y., Valli, M. & Olsen, R. K. Category specificity in the medial temporal lobe: A systematic review. *Hippocampus* **29**, 313–339 (2019).
30. Romo, R., Hernández, A. & Zainos, A. Neuronal Correlates of a Perceptual Decision in Ventral Premotor Cortex. *Neuron* **41**, 165–173 (2004).
31. Broca, P. Remarques sur le siège de la faculté du langage articulé; suivies d'une observation d'aphémie (perte de la parole). *Bull Soc Anthropol.* **36**, 330–357 (1861).
32. Muggleton, N. G., Kalla, R., Juan, C. H. & Walsh, V. Dissociating the contributions of human frontal eye fields and posterior parietal cortex to visual search. *J. Neurophysiol.* **105**, 2891–2896 (2011).
33. Brotchie, P. R. *et al.* Head Position Modulates Activity in the Human Parietal Eye Fields. *Neuroimage* **18**, 178–184 (2003).
34. Nachev, P., Wydell, H., O'neill, K., Husain, M. & Kennard, C. The role of the pre-supplementary motor area in the control of action. *Neuroimage* **36**, 155-63 (2007).
35. Lima, C. F., Krishnan, S. & Scott, S. K. Roles of Supplementary Motor Areas in Auditory

Processing and Auditory Imagery. *Trends Neurosci.* **39**, 527–542 (2016).
36. Mita, A., Mushiake, H., Shima, K., Matsuzaka, Y. & Tanji, J. Interval time coding by neurons in the presupplementary and supplementary motor areas. *Nat. Neurosci.* **12**, 502–507 (2009).
37. Caramazza, A. & Zurif, E. B. Dissociation of algorithmic and heuristic processes in language comprehension: Evidence from aphasia. *Brain Lang.* **3**, 572–582 (1976).
38. Sahin, N. T., Pinker, S., Cash, S. S., Schomer, D. & Halgren, E. Sequential processing of lexical, grammatical, and phonological information within broca's area. *Science.* **326**, 445–449 (2009).
39. Schultz, W., Dayan, P. & Montague, P. R. A neural substrate of prediction and reward. *Science* **275**, 1593–1599 (1997).
40. Bayer, H. M. & Glimcher, P. W. Midbrain dopamine neurons encode a quantitative reward prediction error signal. *Neuron* **47**, 129–141 (2005).
41. Henin, S. *et al.* Learning hierarchical sequence representations across human cortex and hippocampus. *Sci. Adv.* **7**, 4530 (2021).
42. Glasser, M. F. *et al.* A multi-modal parcellation of human cerebral cortex. *Nature* **536**, 171-178 (2016).
43. Rolls, E. T., Huang, C. C., Lin, C. P., Feng, J. & Joliot, M. Automated anatomical labelling atlas 3. *Neuroimage* **206**, 116-189 (2020).
44. Huang, C. C., Rolls, E. T., Feng, J. & Lin, C. P. An extended Human Connectome Project multimodal parcellation atlas of the human cortex and subcortical areas. *Brain Struct. Funct.* **227**, 763-778 (2022).
45. McInnes, L., Healy, J., Saul, N. & Großberger, L. UMAP: Uniform Manifold Approximation and Projection. Preprint at https://arxiv.org/abs/1802.03426 (2018).
