# Supplementary material for: The morphospace of the brain-cognition organisation
Source: Nat Commun. 2024 Sep 30;15:8452. doi: 10.1038/s41467-024-52186-9 (PMC11443123; doi:10.1038/s41467-024-52186-9)
Supplement: Supplementary file 3 — Reporting Summary [file 41467_2024_52186_MOESM3_ESM.pdf]

nature portfolio

Corresponding author(s): Valentina Pacella Michel Thiebaut de Schotten

Last updated by author(s): Jun 7, 2024

Reporting Summary

Nature Portfolio wishes to improve the reproducibility of the work that we publish. This form provides structure for consistency and transparency in reporting. For further information on Nature Portfolio policies, see our Editorial Policies and the Editorial Policy Checklist.

Statistics

For all statistical analyses, confirm that the following items are present in the figure legend, table legend, main text, or Methods section.

n/a	Confirmed	
☐	☒	The exact sample size (*n*) for each experimental group/condition, given as a discrete number and unit of measurement
☐	☒	A statement on whether measurements were taken from distinct samples or whether the same sample was measured repeatedly
☐	☒	The statistical test(s) used AND whether they are one- or two-sided<br>*Only common tests should be described solely by name; describe more complex techniques in the Methods section.*
☐	☒	A description of all covariates tested
☐	☒	A description of any assumptions or corrections, such as tests of normality and adjustment for multiple comparisons
☐	☒	A full description of the statistical parameters including central tendency (e.g. means) or other basic estimates (e.g. regression coefficient) AND variation (e.g. standard deviation) or associated estimates of uncertainty (e.g. confidence intervals)
☐	☒	For null hypothesis testing, the test statistic (e.g. *F*, *t*, *r*) with confidence intervals, effect sizes, degrees of freedom and *P* value noted<br>*Give P values as exact values whenever suitable.*
☒	☐	For Bayesian analysis, information on the choice of priors and Markov chain Monte Carlo settings
☒	☐	For hierarchical and complex designs, identification of the appropriate level for tests and full reporting of outcomes
☐	☒	Estimates of effect sizes (e.g. Cohen's *d*, Pearson's *r*), indicating how they were calculated

Our web collection on statistics for biologists contains articles on many of the points above.

Software and code

Policy information about availability of computer code

Data collection	Meta-analytic maps and single activation maps were manually retrieved.
Data analysis	Data has been analysed via the umap-learn 0.5.1 python 3.4 package, randomise and fslcc tools of FSL 6.0.4 software, Jasper software 0.16.2, FreeSurfer 7.0 software, openly available custom code in python 3.4 that uses matplotlib 3.5.0, nibabel 3.2.1, nilearn 0.7.1, numpy 1.23.5, pandas 2.0.3, scipy 1.10.1, seaborn 0.11.1. The code used in the study is freely available https://github.com/vale-pak/BCS.git.

For manuscripts utilizing custom algorithms or software that are central to the research but not yet described in published literature, software must be made available to editors and reviewers. We strongly encourage code deposition in a community repository (e.g. GitHub). See the Nature Portfolio guidelines for submitting code & software for further information.

Data

Policy information about availability of data

All manuscripts must include a data availability statement. This statement should provide the following information, where applicable:

- Accession codes, unique identifiers, or web links for publicly available datasets
- A description of any restrictions on data availability
- For clinical datasets or third party data, please ensure that the statement adheres to our policy

The dataset analysed during the current study are available at https://www.humanconnectome.org and http://www.Neurosynth.org and at https://github.com/vale-pak/BCS.git.

Research involving human participants, their data, or biological material

Policy information about studies with human participants or human data. See also policy information about sex, gender (identity/presentation), and sexual orientation and race, ethnicity and racism.

Reporting on sex and gender	No sex or gender analysis was carried out, as this study employs functional meta-analytic data.
Reporting on race, ethnicity, or other socially relevant groupings	No race, ethnicity, or other socially relevant groupings analysis was carried out, as this study employs functional meta-analytic data.
Population characteristics	No population characteristic analysis was carried out, as this study employs functional meta-analytic data.
Recruitment	No recruitment was carried out, as this study employs functional meta-analytic data.
Ethics oversight	No ethics oversight was carried out, as this study employs functional meta-analytic data.

Note that full information on the approval of the study protocol must also be provided in the manuscript.

Field-specific reporting

Please select the one below that is the best fit for your research. If you are not sure, read the appropriate sections before making your selection.

- [x] Life sciences
- [ ] Behavioural & social sciences
- [ ] Ecological, evolutionary & environmental sciences

For a reference copy of the document with all sections, see nature.com/documents/nr-reporting-summary-flat.pdf

Life sciences study design

All studies must disclose on these points even when the disclosure is negative.

Sample size	506 meta-analytic maps are part of the experimental dataset available on http://www.Neurosynth.org. The spatial embedding was replicated in four independent out-of-sample datasets: an updated dataset (meta-analytic maps computed after 2017), an external dataset of meta-analytic maps retrieved from https://neuroquery.org/, and single activation maps retrieved from https://neurovault.org/ and https://www.humanconnectome.org/. The four datasets allows to validating the predictive framework under all possible conditions in fMRI studies: two different meta-analytic approaches, contrast-based fMRI results and resting state fMRI.
Data exclusions	No data were excluded from the analyses
Replication	The validation of the study has been performed in four independent datasets.
Randomization	The main analysis of this study is based on meta-analytic data. Validation was conducted on meta-analytic and repositories-based data. The first out-of-sample validation was performed on external individual fMRI data that could not be included in the main analysis due to the different nature of the two datasets. Further out-of-sample validation on another meta-analytic dataset was conducted upon reviewers' suggestions.
Blinding	Blinding is not relevant to the study. The main analysis of this study is based on meta-analytic data. Validation was conducted on meta-analytic and repositories-based data. This is a meta-analysis-based study,

Reporting for specific materials, systems and methods

We require information from authors about some types of materials, experimental systems and methods used in many studies. Here, indicate whether each material, system or method listed is relevant to your study. If you are not sure if a list item applies to your research, read the appropriate section before selecting a response.

Materials & experimental systems

n/a	Involved in the study	
x		Antibodies
x		Eukaryotic cell lines
x		Palaeontology and archaeology
x		Animals and other organisms
x		Clinical data
x		Dual use research of concern
x		Plants

Methods

n/a	Involved in the study	
x		ChIP-seq
x		Flow cytometry
	x	MRI-based neuroimaging

Plants

Seed stocks: n/a

Novel plant genotypes: n/a

Authentication: n/a

Magnetic resonance imaging

Experimental design

Design type: Block Design (but this is a meta-analysis)

Design specifications: N/A (this is a meta-analysis)

Behavioral performance measures: N/A (this is a meta-analysis)

Acquisition

Imaging type(s): Meta-analytic activation maps

Field strength: N/A (this is a meta-analysis)

Sequence & imaging parameters: N/A (this is a meta-analysis)

Area of acquisition: N/A (this is a meta-analysis)

Diffusion MRI ☐ Used ☐ Not used

Preprocessing

Preprocessing software: N/A (this is a meta-analysis)

Normalization: N/A (this is a meta-analysis)

Normalization template: N/A (this is a meta-analysis)

Noise and artifact removal: N/A (this is a meta-analysis)

Volume censoring: N/A (this is a meta-analysis)

Statistical modeling & inference

Model type and settings: Dimensionality reduction and linear regression

Effect(s) tested: n/a

Specify type of analysis: ☒ Whole brain ☐ ROI-based ☐ Both

Statistic type for inference (See Eklund et al. 2016): Voxel-wise

Correction: T-stats resulting from the linear regression were thresholded at p = 0.00033.

Models & analysis

n/a	Involved in the study
☒	☐ Functional and/or effective connectivity
☒	☐ Graph analysis
☐	☒ Multivariate modeling or predictive analysis

Multivariate modeling and predictive analysis	Dimensionality reduction: The Uniform Manifold Approximation and Projection algorithm was used, with of 15 local neighbours to learn the manifold structure of the data points; 0.1 minimum distance was allowed to pack the data; the Euclidean metric was used for the data embedding. Linear regression: Euclidean distances were the dependent variable, and the 505 meta-analytic maps (506 - 1 to-be-predicted map) were the independent variables.
